# Fetal Splenic Artery Pulsatility Index May Predict the Need for Neonatal Intensive Care in Gestational Diabetes Class A1 Cases

**DOI:** 10.3390/jpm14050480

**Published:** 2024-04-29

**Authors:** Mehmet Albayrak, Humeyra Akbas, Emine Seda Guvendag Guven, Suleyman Guven

**Affiliations:** Departments of Obstetrics and Gynecology, Faculty of Medicine, Karadeniz Technical University, Kalkınma Mahallesi Ortahisar, Trabzon 61080, Turkey; mehmetalbayrak@ktu.edu.tr (M.A.); humeyraakbas@ktu.edu.tr (H.A.); emineseda@ktu.edu.tr (E.S.G.G.)

**Keywords:** gestational diabetes, neonatal intensive care unit, prenatal sonography, splenic artery Doppler

## Abstract

The fetal splenic artery pulsatility index is a parameter that reflects fetal well-being and has been used as a predictor of adverse pregnancy outcomes. The aim of this study was to investigate the predictive value of the splenic artery pulsatility index in gestational diabetes mellitus class A1 cases for intensive care unit admission. In this prospective case-controlled study, only sixty single pregnancy cases diagnosed with gestational diabetes mellitus class A1 were evaluated. Fetal splenic artery Doppler parameters such as peak systolic velocity, pulsatility index, resistivity index, and end-diastolic velocity were measured in all cases. The rate of requirements for the neonatal intensive care unit was noted. In cases requiring fetal intensive care, the fetal splenic pulsatility index was found to be statistically significantly lower than in healthy cases without it (0.94 ± 0.29 vs. 1.70 ± 0.53, respectively, *p* < 0.001, Student’s *t*-test). When the fetal splenic PI cutoff value was selected as 1.105 cm^3^, the sensitivity was calculated as 97.9% and the specificity as 58.3% for predicting the need for fetal intensive care (AUC 0.968, *p* < 0.001, 95% CI 0.929–0.998). The use of a low fetal splenic artery PI parameter is a significant and good indicator for predicting the need for fetal intensive care according to the binary logistic regression analysis result (*p* = 0.006). This study suggests that evaluation of fetal splenic artery Doppler in mothers with gestational diabetes mellitus may be used to predict neonates requiring a newborn intensive care unit. Therefore, it is recommended that obstetricians use this simple, rapid, and valuable evaluation of fetal splenic artery Doppler and alert the neonatologist that a newborn intensive care unit may be required.

## 1. Introduction

Gestational diabetes (GDM), a prevalent complication of pregnancy characterized by elevated blood sugar levels, poses significant challenges in managing both maternal and neonatal health [1,2].

Pregnancies complicated by diabetes mellitus, encompassing both gestational and preexisting forms, entail significant risks for maternal and neonatal adverse outcomes. These include higher rates of fetal macrosomia, neonatal hypoglycemia, fetal acidosis, NICU admissions, and cesarean deliveries for fetal distress. The mechanisms underlying these adverse outcomes are multifactorial, potentially involving altered placental vascularization, among other factors [3].

Histologically, placentas from pregnancies complicated by diabetes often exhibit villous immaturity, inflammation, and thickened blood vessel walls [3]. Various pathogenic mechanisms, including tumor necrosis factor-alpha production, hyperinsulinemia, and increased blood viscosity due to hemoglobin glycosylation, contribute to these alterations [4]. Maternal hyperglycemia further stimulates metabolic and hormonal changes in the fetus, increasing fetal oxygen demands and leading to chronic intrauterine hypoxia [5].

Compensatory changes in fetal blood flow, observed through Doppler measurements such as the umbilical artery (UA) and middle cerebral artery (MCA) pulsatility indices, reflect attempts to mitigate the effects of hypoxia [6]. The cerebroplacental ratio (CPR), calculated as the MCA PI to UA PI ratio, serves as a marker of placental blood flow resistance, with decreased CPR indicating chronic hypoxia [4].

Despite the routine implementation of Doppler assessments in high-risk pregnancies, particularly in the third trimester, gaps remain in understanding their implications in pregnancies complicated by diabetes mellitus. 

Fetal Doppler ultrasound emerges as a valuable, non-invasive tool for assessing fetal well-being and detecting potential complications. One such parameter, the fetal splenic artery pulsatility index (PI), derived from Doppler measurements, offers insights into fetal hemodynamics and perfusion. Previous studies have indicated a correlation between abnormal fetal splenic artery PI and adverse neonatal outcomes, particularly in conditions like fetal growth restriction [7].

This prospective study aims to investigate the predictive value of fetal splenic artery Doppler measurements in Class A1 GDM pregnancies regarding the need for neonatal intensive care. By identifying neonates at risk of requiring NICU care, healthcare providers can better prepare and allocate resources, ensuring optimal care for both mothers and infants.

## 2. Materials and Methods

In this study, 75 single pregnancy cases diagnosed with gestational diabetes mellitus class A1 who applied to our high-risk pregnancy outpatient clinic were evaluated over a one-year period. Ethics committee approval was obtained. 

The study groups were aged 18–40 years, who presented at the polyclinic for routine antenatal follow-up in the third trimester of pregnancy (>28 weeks) and had the diagnosis of gestational diabetes class A1. Only the pregnant women at 30–34 weeks of gestation (including the limit weeks) were included in the study. In the first examination of the pregnant cases, it was determined that they did not contain any high-risk pregnancy factors other than gestational diabetes, and liver, kidney, thyroid, and hematological test results were within normal limits. A diagnosis of gestational diabetes mellitus (GDM) was made with a 75 g oral glucose tolerance test (OGTT). The 75 g OGTT was used for diagnosing diabetes in pregnant individuals. The two-hour 75 g OGTT was diagnostic of GDM when one glucose value was elevated. According to this test, fasting blood glucose was measured following an 8 h fasting period, and then a solution containing 75 g of glucose was drunk. Blood glucose was measured 1 h and two hours after drinking the solution. GDM was diagnosed when fasting blood glucose was 92, first-hour blood glucose was 180, or second-hour blood glucose was 153 mg/dL and above. Only the cases that were euglycemic with diet were accepted as GDM Class A1 [8].

Cases with any systemic disease, use of drugs other than vitamins and iron, multiple pregnancies, smoking, biochemical/hormonal/hematological test disorders, and cases diagnosed with congenital fetal anomalies were not included in the study. Cases with abnormal uterine, umbilical, and middle cerebral artery Doppler findings were not included in the study. Fifteen cases not meeting the inclusion criteria were excluded from the study. Eleven cases who did not come for pregnancy follow-ups and four cases who developed any pregnancy complications were excluded from the study.

In the study, all participating women provided informed consent. Key information collected included age, gravidity, parity, and body mass index (BMI), as well as sonographic measurements of fetal biometry, such as biparietal diameter (BPD), abdominal circumference (AC), femur length (FL), and estimated fetal weight (EFW). The gestational age was verified using first-trimester ultrasound data.

**Figure 1:**
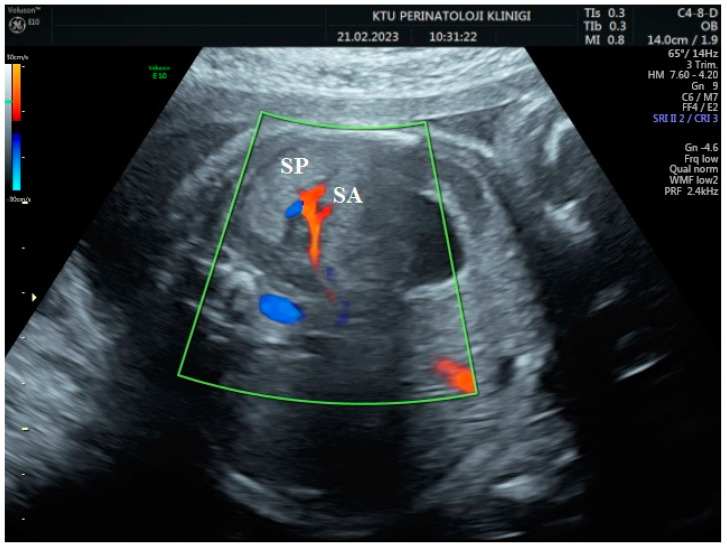
Two-dimensional ultrasonographic imaging of the 30-week-old fetus shows the fetal spleen (SP) and splenic artery (SA).

**Figure 2:**
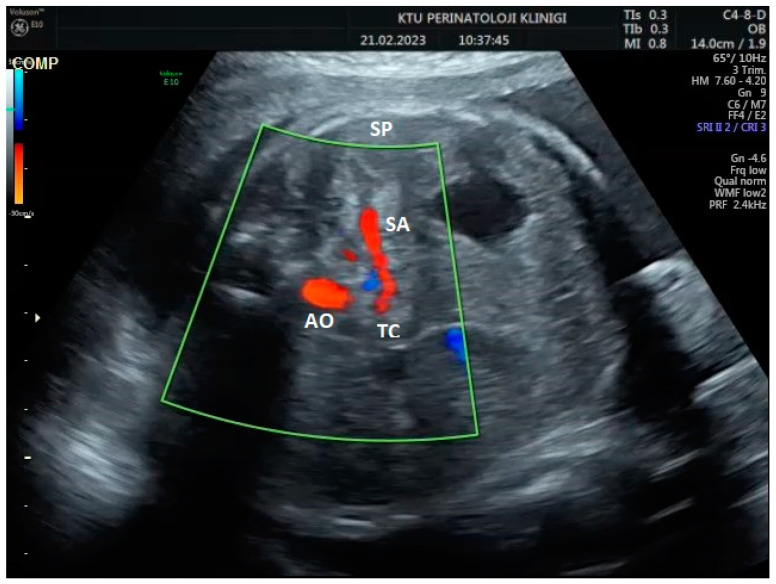
Two-dimensional ultrasonographic imaging in the axial plane of the 30-week-old fetus shows the origin of the fetal splenic artery from truncus coeliacus (TC; truncus coeliacus, AO; aorta, SA; splenic artery, SP; fetal spleen).

**Figure 3:**
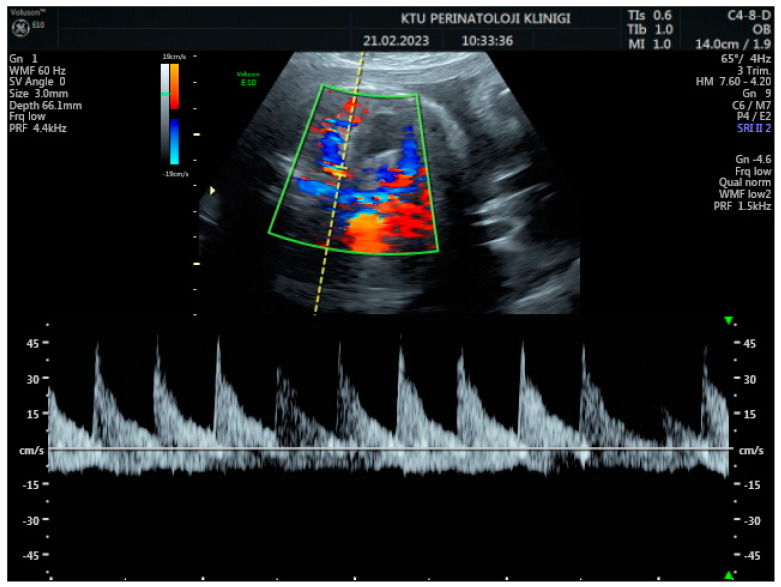
Two-dimensional ultrasonographic imaging of the 30-week-old fetus shows pulsed Doppler waveforms of the splenic artery.

During the Doppler assessment, the patient was positioned on the examination table in a supine posture. It was measured when the fetus was inactive. A standard AC axial section view was obtained. The stomach pocket was visualized. A color Doppler was placed on the upper part of the stomach pocket, and the splenic artery was visualized at the entrance to the spleen. Later, it was observed that the trace progressed towards the truncus, celiacus, and abdominal aorta. A color Doppler was placed at the entrance to the spleen with a 0-degree insomination angle without the splenic artery entering the spleen. Fetal splenic artery Doppler parameters such as peak systolic velocity (PSV), pulsatility index (PI), resistivity index (RI), and end-diastolic velocity (EDV) were measured in all cases (Figure 1, Figure 2 and Figure 3). Following fetal splenic artery imaging, the vessel diameter was measured from the area directly in the middle. The vessel measurement was made from outside to outside, and the insomination angle was adjusted to be <30 degrees.

All sonographic measurements were performed via the 2016 model VOLUSON GE E10 sonograph unit (GE Penta Electronic and Medical System Co., Istanbul, Turkey). All sonographic measurements were made at least twice by an experienced clinician. All cases were followed up until the end of pregnancy, and the birth date of the mother and the baby (fetal birthweight, fetal umbilical cord blood gas analysis) and the need for neonatal intensive care were recorded.

A total of 12 cases required neonatal intensive care due to respiratory distress, and their data were compared to those not needing intensive care.

### 2.1. Statistical Analysis

The data were analyzed using SPSS 13 software. The Levene test assessed variance homogeneity, while the Kolmogorov–Smirnov test evaluated normal distribution compatibility. Chi-square and Student’s *t*-tests were employed for statistical analysis. To identify fetal splenic artery Doppler parameters for predicting neonatal intensive care requirements, ROC analysis was conducted. All data are presented as the mean ± standard deviation or percentage. The ability of fetal Doppler parameters to predict neonatal intensive care needs was assessed using binary logistic regression analysis. 

The correlation between the parameters was evaluated with the help of the Pearson correlation test. The situation where the *p*-value was below 0.05 was considered significant.

### 2.2. Ethical Approval

The study protocol was approved by the current university ethics board (number 2020/37, date 28 January 2020).

## 3. Results

The data from 60 cases in total were evaluated. The demographic and sonographic factors of cases who need fetal intensive care and those who do not are shown in Table 1. 

**Figure 4:**
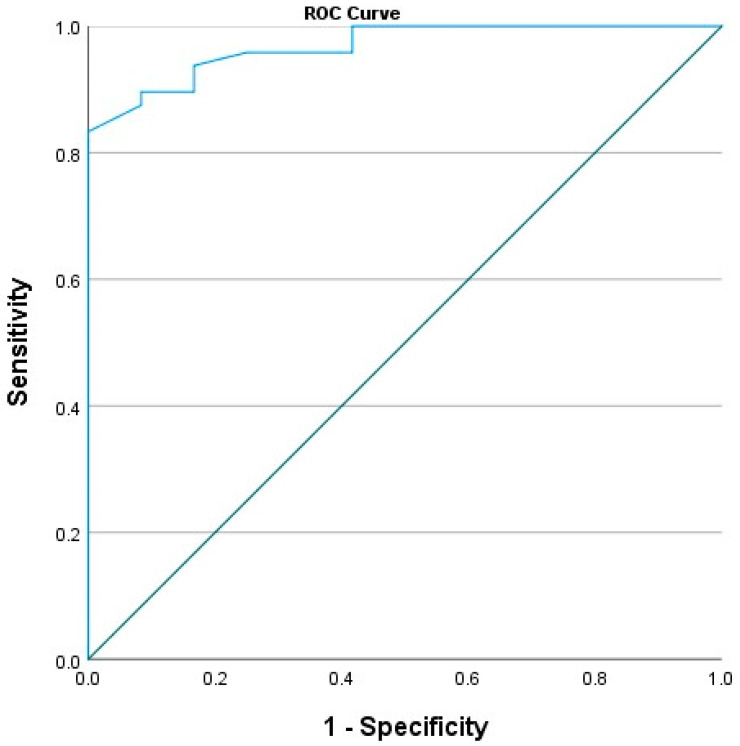
Receiver operating curve analysis data showing the relationship between the calculated fetal splenic artery PI value and the need for neonatal intensive care are presented.

In cases requiring fetal intensive care, the fetal splenic PI index was found to be statistically significantly lower than in healthy cases without it (0.94 ± 0.29 vs. 1.70 ± 0.53, respectively, *p* < 0.001, Student-*t* test). When the fetal splenic PI cutoff value was selected as 1.105, the sensitivity was calculated as 97.9% and the specificity as 58.3% for predicting the need for fetal intensive care (AUC 0.968, *p* < 0.001, 95% CI 0.929–0.998, Figure 4). The use of a low fetal splenic artery PI parameter is a significant and good indicator for predicting the need for fetal intensive care, according to the binary logistic regression analysis result (*p* = 0.006). Pearson correlation analysis results revealed that a moderate positive correlation was found in terms of fetal umbilical cord pH and BE at delivery and prenatal fetal splenic Doppler PI in all cases (Pearson correlation, r = 0.334, *p* = 0.009, Pearson correlation test). 

## 4. Discussion

This study evaluated the relationship between fetal splenic artery Doppler parameters and the need for a NICU in pregnant women with GDM class A1. The results showed that a lower fetal splenic artery PI was significantly associated with an increased need for NICU, with a sensitivity of 97.9% and a specificity of 58.3% for predicting the need for neonatal intensive care when the fetal splenic PI was taken as 1.105. The results also showed a moderately positive correlation between prenatal fetal splenic Doppler PI and fetal umbilical cord pH and BE at delivery.

Pregnancies complicated by diabetes mellitus, including gestational diabetes, are associated with increased risks of adverse perinatal outcomes. These risks can be mitigated through enhanced monitoring and informed decision-making regarding the timing of birth. The 36-week ultrasound is particularly critical as it provides essential data for decision-making in the late stages of pregnancy. However, the clinical implications of abnormal Doppler findings in such pregnancies are not well understood, underscoring the need for clear guidelines and threshold values to identify and manage at-risk pregnancies effectively [7].

The UA Doppler is the most universally utilized Doppler measurement due to its simplicity and low skill requirement. It plays a crucial role in assessing the well-being of the fetus by measuring blood flow in the umbilical artery. This Doppler measure is particularly valuable in pregnancies complicated by diabetes mellitus, where it has shown superior prognostic accuracy for predicting adverse perinatal outcomes compared to other Doppler measures [7].

The MCA Doppler and CPR have been proposed as additional tests to enhance fetal assessment in diabetic pregnancies. However, these measures are technically challenging and difficult to obtain accurately in later pregnancy. The review findings indicate that the prognostic accuracy of the UA Doppler outperforms that of the MCA Doppler and CPR in diabetic pregnancies. The lower prognostic accuracy of CPR, compared to UA and MCA Dopplers, may be attributed to the fact that most available data on CPR’s predictive ability are derived from cohorts of preterm pregnancies complicated by growth restriction. Given the complexities and technical challenges associated with MCA Doppler and CPR, it is recommended that the UA Doppler remain the primary Doppler assessment tool in pregnancies complicated by diabetes mellitus [7].

The fetal spleen plays a crucial role in hematopoiesis until the late fetal period [9]. Typically not visible on ultrasound before 18 weeks, its prominence increases in cases of fetal anemia or intrauterine infection due to extramedullary hematopoiesis [10]. Studies have explored the spleen’s size and volume in relation to gestational age, suggesting normal values for the diameters of the fetal spleen. This correlation between splenic size and various hematologic, infectious, and developmental syndromes underscores the clinical importance of establishing normal values for the fetal spleen [10].

Doppler measures of the splenic artery have therapeutic relevance, especially in diagnosing fetal conditions such as growth restriction and anemia [11]. The splenic artery, originating from the celiac trunk and traveling behind the stomach to the spleen, can be assessed for peak systolic velocity (PSV) and pulsatility indices (PI). The first systematic longitudinal study by Ebbing et al. provided reference values for these measures, highlighting the utility of splenic artery Doppler velocimetry as an additional diagnostic tool [12].

In prenatal care, integrating splenic artery Doppler measures can enhance the diagnosis and management of fetal anemia or intrauterine infection [13,14]. The correlation between the splenic artery’s flow velocity and pulse with fetal growth restriction and anemia further supports the use of Doppler velocimetry of the splenic artery in establishing or confirming a diagnosis [15]. Additionally, the study by Ebbing et al. demonstrated a correlation between the umbilical venous supply to the right liver lobe and portocaval pressure, as well as splenic and celiac artery flow, offering insights into differentiating between splanchnic and fetal circulation [12].

The importance of fetal Doppler parameters in predicting adverse neonatal outcomes has been well documented in the literature [16,17]. Previous studies have focused on umbilical and middle cerebral artery Doppler measurements in high-risk pregnancies, including those with GDM [4,7]. Limited research has been conducted on the relationship between fetal splenic artery Doppler parameters and neonatal outcomes in pregnancies complicated by GDM class A1. In the past decade, abnormal Doppler velocity patterns in various fetal vessels and their correlation with fetal outcomes have been extensively studied [18,19]. Recently, the pathophysiology of blood redistribution in relation to fetal growth deprivation has been evaluated [20]. Epidemiological evidence suggests that changes in blood distribution due to fetal growth deprivation may increase the risk of myocardial damage and abnormal liver function in newborns [20]. Decreased resistance in the splenic artery has been suggested as an indicator of fetal distress, and decreased fetal liver circulation has recently been proposed as a predictor for the prognosis of fetal growth restriction [20]. 

The assessment of fetal well-being is crucial in prenatal care, particularly for high-risk pregnancies. Fetal splenic artery Doppler ultrasonography is a non-invasive technique that has been increasingly utilized for evaluating fetal well-being and predicting adverse perinatal outcomes. The technique measures blood flow velocities in various vessels, including the splenic artery, which supplies blood to the spleen, an organ essential for fetal immune system development. The Doppler indices, such as resistance index (RI), pulsatility index (PI), and systolic/diastolic ratio (S/D), can help assess blood flow and detect abnormalities in fetal circulation [11,15].

Abuhamad et al. (1992) were the first to report the use of Doppler velocimetry in evaluating the main splenic artery of fetuses [11]. They noted a reduction in the splenic artery RI in a subset of fetuses classified as small for gestational age (SGA). The authors theorized that the associated chronic hypoxia led to the release of erythropoietin, which increased splenic flow to support increased erythropoiesis. This reduction in RI in some SGA fetuses was later confirmed by Capponi et al. in a subsequent study [16]. 

Several theories attempt to explain the reduced splenic artery pulsatility index (PI) observed in cases of fetal growth restriction. Recent research has shown that compensatory vasodilation in the splenic artery helps maintain low venous perfusion to the fetal liver [20]. The exact connection between a decreased splenic artery PI and fetal growth restriction remains unclear. However, one study discovered that a lower splenic artery PI was correlated with higher perinatal mortality rates, lower Apgar scores, and metabolic acidosis. This suggests that severe fetal deprivation may lead to more significant hemodynamic changes in the spleen, potentially identifying fetuses at an increased risk of perinatal death [21].

In a study, it was determined that fetal splenic artery PI values were lower in fetuses small for gestational age compared to normal fetuses. This supports the argument that vascular resistance is lower in SGA fetuses [11]. 

In another study, fetal splenic artery Doppler measurement was performed in pregnancies with late-onset fetal growth restriction, and similar results were reported. The researchers found that decreased splenic artery PI was significantly and positively correlated with a higher probability of experiencing adverse obstetric outcomes [15].

Neonatal outcomes of splenic artery Dopplers in gestational diabetic patients have not yet been studied. As a result, our study is the first of its kind in the literature. The current study demonstrates that fetal splenic artery PI could serve as a valuable indicator for predicting the need for neonatal intensive care in GDM class A1 pregnancies. A possible explanation for this finding could be that a lower PI reflects increased placental resistance and, consequently, impaired fetal blood flow, which could negatively impact fetal growth and well-being. This notion is supported by the observed moderately positive correlation between prenatal fetal splenic Doppler PI and fetal umbilical cord pH and BE at delivery, suggesting a relationship between splenic artery PI and fetal acid-base status.

The results of this study are consistent with previous findings that have demonstrated the utility of fetal Doppler measurements in predicting neonatal outcomes in high-risk pregnancies. However, the current study adds to the existing body of knowledge by specifically focusing on GDM class A1 pregnancies and examining the association between fetal splenic artery Doppler parameters and neonatal outcomes.

The underlying mechanisms by which a lower fetal splenic artery PI may predict the need for NICU admission in GDM class A1 cases are not fully elucidated by the current study. However, several potential pathways can be hypothesized based on the existing literature. One possible explanation is that the alterations in the splenic artery Doppler waveform reflect a redistribution of fetal blood flow in response to placental insufficiency or other stressors associated with maternal diabetes. In this scenario, the fetus may prioritize perfusion to vital organs, such as the brain, at the expense of less essential vascular beds like the spleen. This “brain-sparing” effect, while initially protective, may ultimately lead to fetal compromise and the need for NICU care if the placental dysfunction progresses. 

Additionally, the splenic artery Doppler may provide insights into the fetal inflammatory and immune responses to the metabolic derangements associated with maternal diabetes. The spleen is an important lymphoid organ involved in the body’s immune response, and alterations in its blood supply may reflect the fetus’s ability to mount an appropriate inflammatory and immune reaction to the diabetic intrauterine environment. Impaired splenic perfusion, as indicated by a lower PI, may be a marker of the fetus’s inability to effectively respond to the metabolic and vascular stressors of maternal diabetes, ultimately leading to an increased risk of adverse neonatal outcomes and the need for NICU admission. From a clinical perspective, the findings of this study suggest that the evaluation of the fetal splenic artery Doppler, particularly the pulsatility index, may be a valuable addition to the assessment of fetal well-being in pregnancies complicated by GDM class A1. This novel Doppler parameter could potentially be used in conjunction with traditional Doppler assessments of the umbilical and uterine arteries to provide a more comprehensive evaluation of fetal hemodynamics and identify fetuses at higher risk of requiring NICU care. 

The high sensitivity of the splenic artery PI cutoff value (1.105) for predicting NICU admission suggests that this parameter could be used as a screening tool to identify high-risk cases that may benefit from more frequent fetal monitoring, earlier delivery, or other interventions to optimize neonatal outcomes. 

In this study, low umbilical pH and BE values were found to be correlated with low splenic artery PI values. There were no signs or symptoms of fetal distress in any of the cases in this study. The reason for such a correlation may be the vascular constriction and temporary endothelial damage caused by gestational diabetes in all fetal vessels (including the splenic artery). In cases that cannot overcome this possible temporary damage process after birth, fetal intensive care is needed, just like in IUGR and fetal distress cases. However, research involving large-scale laboratory and molecular studies is needed to test this hypothesis.

Technically, for measurement, the fetus’s stomach should be at 12 o’clock, and the spine should be at 9 o’clock. It is not always possible to catch the fetus in this position. If the fetus is not in this position during the measurement, we can recommend that the mother eat and walk around for a few hours. It could be measured after a few hours, or it could be tried to turn the fetus in the abdomen with the two-hand technique. In this research, it was not possible to find an appropriate position in four cases. With these two techniques, the appropriate position was then achieved.

Measurements for all cases were made in the third trimester. Measurements were taken at an average of 31–32 weeks in both groups. As it is known, complications such as IUGR, macrosomia, and fetal growth disorders in gestational diabetes begin after the 28th week of gestation. There are not enough literature data as to why it starts this week. For this reason, the research design was planned based on the measurements taken after these weeks. However, it is not clear in the literature which week is best to measure. In addition, according to our preliminary study results, splenic artery visualization after 28 weeks was preferred this week because it will be sonographically easier and will provide more accurate and objective measurement results. In our preliminary study, single measurement values were presented because there was no significant change in splenic Doppler on a week-by-week basis during the follow-up period until birth in a few cases. Making a single measurement and taking measurements after the 28th week are the limitations of this research in this regard.

### Study Limitations 

The current study has several limitations that should be considered when interpreting the results. Firstly, the sample size, while adequate for the statistical analyses performed, was relatively small, with only 12 cases requiring NICU admission. The most important limitation of our study was that it included 12 cases and 48 control cases. Future studies with large case and control series may confirm our results. Larger, multicenter studies are needed to confirm the findings and further elucidate the role of the fetal splenic artery Doppler in predicting adverse outcomes in GDM class A1 pregnancies.

Additionally, this study was limited to a specific gestational age range (30–34 weeks) and did not evaluate the longitudinal changes in the splenic artery Doppler throughout pregnancy. It is possible that the predictive value of this parameter may vary depending on the stage of gestation, and future studies should explore the temporal dynamics of the splenic artery Doppler in diabetic pregnancies.

Another limitation is the lack of long-term follow-up data on the neonatal and childhood outcomes of the study participants. It would be valuable to investigate whether the alterations in the fetal splenic artery Doppler are associated with not only the need for NICU admission but also with more long-term developmental and health outcomes. Future research should also aim to elucidate the underlying mechanisms by which the fetal splenic artery Doppler reflects fetal well-being in the context of maternal diabetes. Correlative studies with other markers of fetal compromise, such as biochemical indicators of oxidative stress or inflammation, may provide further insights into the pathophysiological pathways involved.

Additionally, it would be interesting to explore the potential utility of the fetal splenic artery Doppler in the prediction and management of other pregnancy complications associated with maternal diabetes, such as fetal macrosomia, shoulder dystocia, and neonatal hypoglycemia. The splenic artery Doppler may offer unique insights into fetal adaptations to the diabetic intrauterine environment that are not captured by traditional Doppler assessments.

## 5. Conclusions

Based on this study’s results, it is believed that using fetal splenic artery Doppler measurements in mothers with gestational diabetes mellitus (GDM) can help predict which neonates may require a NICU. We aimed to determine the best method for predicting NICU needs for infants born to mothers with GDM. As this study suggests, the evaluation of fetal splenic artery Doppler could be a brief and valuable answer to this question.

The current researcher kindly recommended this simple, rapid, and valuable evaluation of fetal splenic artery Doppler to obstetricians. By doing so, it may be a safe approach to alert the neonatologist that a NICU might be required, allowing for better preparedness and resource allocation.

The results of this study suggest that fetal splenic artery PI could be a valuable indicator for predicting the need for neonatal intensive care in pregnant women with GDM class A1. This finding could have potential clinical implications, as it may help guide prenatal care and counseling for women with GDM, thereby potentially improving neonatal outcomes. Further research is needed to confirm and extend these findings in larger populations and to explore the long-term neonatal outcomes associated with fetal splenic artery Doppler parameters.

## Figures and Tables

**Table 1:** The demographic and sonographic factors of cases who had the need for neonatal intensive care and who did not.

Demographic/Sonographic Factors	Need for Neonatal İntensive Care (n = 12)	No need for Neonatal İntensive Care (n = 48)	*p*-Value
Age (years)	32.17 ± 4.91	31.38 ± 5.01	0.627
Gravida (no.)	3.00 ± 2.09	2.81 ± 1.62	0.737
Parity (no.)	2.50 ± 1.69	1.59 ± 1.19	0.078
Body mass index (kg/m^2^)	34.11 ± 8.85	31.22 ± 5.65	0.166
Gestational week at measurement	32.73 ± 0.94	32.30 ± 1.14	0.227
EFW at measurement (gr)	2176.75 ± 627.65	2186.42 ± 636.63	0.963
Gestational week at birth	38.25 ± 1.14	38.02 ± 2.12	0.715
Fetal splenic artery PSV	34.58 ± 9.04	37.02 ± 14.78	0.587
Fetal splenic artery S/D	4.16 ± 0.49	4.23 ± 1.68	0.796
Fetal splenic artery PI	0.94 ± 0.29	1.70 ± 0.53	<0.001
Fetal splenic artery RI	0.67 ± 0.20	0.75 ± 0.20	0.260
Fetal splenic artery diameter	0.40 ± 0.26	0.49 ± 0.63	0.636
Caesarean section delivery (%)	41.7% (5/12)	41.7% (20/48)	0.624 ^a^
Fetal gender (F/M)	5/7	23/25	0.467 ^a^
Infant birthweight (gr.)	3397.08 ± 516.25	3195.00 ± 577.31	0.273
Fetal umbilical artery pH	7.28 ± 0.40	7.36 ± 0.04	<0.001
Fetal umbilical artery BE	−1.34 ± 1.36	−1.18 ± 1.81	0.782
APGAR scored in the first minute	6.33 ± 0.65	6.79 ± 1.35	0.188
AGRAR scored in the fifth minute	8.25 ± 0.97	8.50 ± 0.77	0.344

PSV: Peak systolic velocity; S/D: Systol/Diastole; PI: Pulsatility index; RI: Resistivity index; BE: Base excess. The Student-*t* test and ^a^ Fisher’s exact chi-square tests were used in the statistical analyses. Data are presented as the mean ± standard deviation values or as the number of cases and percentage.

## Data Availability

Any data set is available on request to the corresponding author.
